# Thank you to *The Lancet Regional Health – Europe's* clinical and statistical peer reviewers in 2024

**DOI:** 10.1016/j.lanepe.2025.101232

**Published:** 2025-02-03

**Authors:** Pooja Jha, Rafael Cardoso, Ivana Nedic, Stephanie Becker, Heather Brown

**Affiliations:** The Lancet Regional Health – Europe

In **February 2023, we pledged** to enhance the diversity, transparency, and inclusivity of our peer review process while continuing to uphold the rigorous standards that define *The Lancet Regional Health – Europe*. As we reflect on the year 2024, we are pleased to report the progress toward these goals, while also identifying areas for further improvement. This progress would not have been possible without the dedication of our peer and statistical reviewers, whose critical contributions have been vital to maintaining the quality of our publications.

In 2024, 1192 reviewers provided their expertise, marking a 30% increase compared to 2023. This substantial growth reflects our journal's expanding reach and the continued trust placed in us by the global research community.

**Gender diversity** in reviewer demographics remains a cornerstone of our efforts. In 2024, 56.7% of reviewers identified as men (down from 57.8% in 2023), 34.6% identified as women (up from 32.5%), 0.8% identified as non-binary or gender diverse (up from 0.5%), and 8.0% chose not to disclose their gender identity (down from 9.2%). While we are pleased with progress, we recognise the need to further improve gender diversity, including reducing the 22% gap between reviewers who identify as men and those who identify as women, and we are dedicated to addressing this disparity in the years to come.

**In terms of geographical representation**, while the majority of our reviewers (93.2%) were from high-income countries (HICs), we are encouraged to see contributions from low- and middle-income countries (LMICs) increase to 5.5% from 4.8% in 2023. This remains an area where we are committed to fostering further engagement and inclusivity. For 1.3% of reviewers, country information was unavailable, a slight improvement from 2023, where 2.1% of reviewers did not provide their country data. We aim to continue refining our data collection.

Our reviewers came from 55 countries, underscoring the international collaboration that defines research. The largest numbers of reviewers came from the United Kingdom (197), the United States (150), Italy (120), and Germany (107). We will strive to intensify efforts to have reviewers from underrepresented regions in 2025.

**Reflecting on our 2023 pledges**, we have made measurable progress in several key areas, including expanding our reviewer pool, enhancing global representation, narrowing the gender gap, and increasing representation from LMICs. However, there is still work to be done to further close the gaps in all these vital aspects in the years ahead.

A significant initiative across all Lancet journals has been the update to our Editorial Manager submission portal with Equity, Diversity, and Inclusion (EDI) personal classifications, launched in September 2024. Users can now voluntarily assign these EDI classifications to submitted manuscripts or to their individual accounts to indicate their expertise. We anticipate that this feature will enhance reviewer searches and contribute to our broader diversity and inclusion goals.

As we move into 2025, our focus will remain on broadening the diversity of our reviewer pool. Once again, we extend our heartfelt gratitude to all our reviewers for their time, expertise, and dedication in 2024. Your efforts ensure that The Lancet Regional Health – Europe continues to publish impactful, high-quality research that drives meaningful change in health outcomes across Europe and beyond.

Armando Abergel

Karim Abu-Omar

Tim Adair

Banwari Agarwal

Ajay Aggarwal

Antonella Agodi

Charles Agyemang

Viktor Ahlqvist

Mohamed Ahmed

Raheel Ahmed

Sofia Ahmed

Leman Akcan Yildiz

Angela Alberga

Jennifer Albrecht

Werner Albrich

Mohammed Ali Al-Garadi

Rustam Al-Shahi Salman

Antonella Amendola

Faisal Mohammad Amin

Jacob Amir

Antonio J Amor

Michael Anderson

Nicolaus Andratschke

Stefan Andreas

Andrea Angheben

Spinello Antinori

Jean François Argacha

Gema Ariceta

Elizabeth V Arkema

L Eugene Arnold

Diane A I Ashiru-Oredope

Helena Askling

Francesco Asnicar

María Pilar Astier-Peña

Cécile Aubron

Kristin Aunan

Dagfinn Aune

Marianne Aznar

Louise Baandrup

Marc F D Baay

Rafael Badenes

Laura Baena-García

Anees Bahji

Jinbing Bai

Shasha Bai

Giancarlo Balercia

Francisco Javier Ballesteros-Rodriguez

Lavinia Baltes-Flueckiger

Amitava Banerjee

Puneet Bansal

Maggie Banys-Paluchowski

Yanping Bao

Eugenio Baraldi

Louise Barbier

Martina Barchitta

Ruanne Barnabas

Andrew James Barnes

Alfred Bartolucci

Stefania Basili

Dirk Bassler

Anna Basu

Partha Sarathi Basu

Salvatore Battaglia

David Baud

Nicole Baumann

Sina Bavari

Antonios Bayas

Hasan Bayram

Peter Becher

Jasper Been

Paul Beggs

Josip Begovac

Brandon K Bellows

Sergi Beltran

Andréa L Benedet

Christian Benedict

Caue Benito Scarim

Peter Bentzer

Beatrice Bercot Bercot

Elling Tufte Bere

Mette M Berger

Niels Bergsland

Giorgi Beridze

Peter Berlit

Anne H Berman

Teresa Bernal

Eduardo Berriatua

Riccardo G Bertolo

Astrid Bertsche

Giulia Berzero

Mael Bessaud

Marc Besselink

Sanjay Bhagani

Amarnath Bhide

Angelo Bianchetti

Bart Biemond

Isobel Blake

Flora Blangis

Torsten Gerriet Blum

Stefania Boccia

William Boden

Laura Boekel

Cornelis Boersma

Marialaura Bonaccio

Francesco Bonella

Petri Bono

Gouke Bonsel

Louis Bont

Rohan Borschmann

Stephan Bose-O'Reilly

Edoardo Botteri

Kathryn H Bowles

Kayvan Bozorgmehr

Tilman Brand

Axel Brandes

Giampaolo Brichetto

Bianca Brijnath

Amy Brodtmann

Benjamin Brokinkel

Roger L Brown

Ronny Bruffaerts

Nathan E Brummel

Fabrizio Bruschi

Raffaele Brustia

Udo Buchholz

Joshua Buckman

Pierre A Buffet

Raffaele Bugiardini

Thomas Bunch

Danilo Buonsenso

Johan Burisch

Patrizia Burra

Christian Burri

Christopher Burton

Christopher Butler

Jane A Buxton

Paul Bywaters

Joaquin Cabezas

André Cabié

Carlo Caffarelli

Banu Çakir

Russell Clarence Callaghan

Michael Calnan

Andrea Camerini

Christine Campbell

Marjo Campmans-Kuijpers

Daniela Canella

Javier Cañueto

Joseph Cappelleri

Gaetano Caramori

Verena Carrara

Claire Carson

Jeffrey Carson

Ben Carter

Jean-Sebastien Casalegno

Maria Casanova

Antonio Cascio

Marco Cassone

Manuel Castro Cabezas

Juan Cata

Boudewijn Catry

Sandro Cattacin

Giulia Cavestro

Paolo Cavoretto

Maurizio Cecconi

Luca Cegolon

Leo Anthony Celi

Jad Chahoud

Martin Chalumeau

Sherry Kit Wa Chan

Yoke Fun Chan

Wing Chung Chang

Nicholas Charron

Denise A Chavira

Bo Chawes

Marcus Chen

Yingyao Chen

Zhen Chen

Wei Cheng

Jeanie Cheong

Bernard MY Cheung

Li Cheung

Tanuja Chitnis

Davide Chiumello

Brian C Cho

Kelvin Choi

Peter Christensen

Robert Christensen

Hsiao-Chi Chuang

Irfan Cicin

Juan-Carlos Císcar

Olivier Claris

Loa Clausen

Marie-Annick Clavel

Tammy Clifford

Adam Fa Cohen

Intira Collins

Christian Combe

Blandine Comte

Tim Cooksley

Oliver Cornely

Eva Corpeleijn

Giulia Corrao

Chiara Corti

Paul Cottu

Anthony Cousien

Laura Cowley

Susanna Cramb

Timothy Cross

Louise Crowe

Jennifer Cuellar-Rodriguez

Chris Cunningham

Ruth Cunningham

Malcolm Cutchin

Kamila Czene

Anabela Gonçalves da Silva

Omid Dadras

Antonio D'Alessio

Emanuele D'Amico

Gheorghe Andrei Dan

Rolando Maria D'Angelillo

Anna-Karin Danielsson

Carl K D'Arcy

Prokar Dasgupta

Shouro Dasgupta

Shanlee M Davis

Lynne Dawkins

Fatimah Dawood

Andrew Day

Rob De Bie

Jana de Boniface

Giovanni de Gaetano

Xavier de Lamballerie

Luca De Nicola

Anita De Rossi

Anniek de Ruijter

Nipun Lakshitha de Silva

Adam de Smith

Maartje De Wit

Olivier de Witte

Geoffrey Debelle

Joost Dekker

Annarosa Del Mistro

Marco Del Riccio

Christophe Delclaux

Marcos DelPozo-Baños

Claire Demoury

Saskia den Boon

Hansen Deng

Petra Denig

Payal Desai

Emmanuel Deshayes

Marie Desnos-Ollivier

Guenther Deuschl

Delan Devakumar

J Hans DeVries

Gabriella Di Giuseppe

Esperanza Diaz Perez

Igor Diemberger

Rodrigo Dienstmann

Pablo Díez-Villanueva

Guodong Ding

Mara Dinsmoor

Lien Anh Ha Do

Christian Dobel

Matthew Dodd

Richard Dodel

Jens Dollerup

Alexander Domnich

Lorie Donelle

Maria Dorobantu

Abigail Dove

Jenny Downs

Silvano Dragonieri

Stephanie Dramburg

Janine Dretzke

Jelena Drulović

Xianglin Du

Raffaele Dubbioso

Ralf Dürrwald

Jennifer Dykxhoorn

Brian Earp

Marloes Eeftens

Terje Eikemo

Luca Elli

Sascha Ellington

Rami El-Shafie

Jon Ivar Elstad

Louise Emilsson

Jean-Philippe Empana

Bo Engdahl

Charli Eriksson

Paolo Eusebi

Frank Euteneuer

David Ewing

Antonio Facciorusso

Fadi Fakhouri

Henrik Falconer

Christiane Falge

Amary Fall

Yiru Fang

Fabio Farinati

Michelle Farrar

Forough Farrokhyar

Laurent Fauchier

Seena Fazel

Óscar Fernández

Josep Figueras

Jordi Figuerola

Filippos T Filippidis

Cristian Fiori

Benedikt Fischer

Florian Fischer

Kelly Flemming

Guillaume Fond

Arnaud Fontanet

Nathan P Ford

Karin M E Forsberg

Asta Forsti

Sandrine Fosse-Edorh

Caroline Foster

David Foxe

Francesco Franceschi

Samuel A Frank

Kristian Steen Frederiksen

Mark Freedman

Jennifer Freeman

Nicholas Freemantle

Heinz Freisling

Neil French

Emilie Friberg

Signe Friesland

Giovanni Frisoni

Julie Fritz

Benedikt Fritzsching

Ole Frobert

Gary Frost

Lars Frost

Elaine Fuertes

Ruben Georges Fukkink

Mathurin Fumery

Roberto Furlan

Daniel G Abiétar

Rhian Gabe

Albis Francesco Gabrielli

Lorenzo Gaetani

Larissa Gaias

María José Galindo

Peter Galle

Marco Gallo

James Galloway

Silvano Gallus

Trivadi Ganesan

Junyi Gao

Xiang Gao

José Manuel García Domínguez

Clemente Garcia-Rizo

Bradley Gardiner

Pietro Gareri

Claire V Garnett

Amalia Gastaldelli

James Gaughan

Anna Geldermann

Marc Gelkopf

Ivan Gentile

Marco Geraci

Vania Giacomet

Anna Maria Giammarioli

Diana Giannarelli

Duncan Gilbert

Felix Gille

Marta Giovanetti

Philippe Girard

Paolo Girardi

Matthias Girndt

Rebecca Gladstone

Federico Gobbi

Shira Goldenberg

Krzysztof Goniewicz

Benjamin Goodair

Shinya Goto

Rebecca Gottesman

Claire Goumard

Igor Grabovac

Yonatan Grad

Cristina Granja

Jakob Grauslund

William Gray

Lorraine Greaves

Michael James Green

Jonathan Grigg

Pavel Grigoriev

Harry Groen

Peter Groenewegen

Henning Grønbæk

Anders Grøntved

Dirk Grunhagen

Viktor Grünwald

Maria Guarino

Martin Gulliford

Marc Gunter

Stefan Gutwinski

Christine Gyldenkerne

Allan Hackshaw

Riad Haddad

Gertrud Hafstad

Tim Hagenacker

Omar Hahad

André Hajek

Georg Halbeisen

William Haley

Per Hall

Sarah Halligan

Steve Halligan

Haitham Hamoda

Paul Hanly

Biswadjiet Harhangi

Neil Harrison

Katie Harron

Jaime Hart

William Hart

Martin Härter

Cesare Hassan

Claire Hastie

Martin Hausberg

Joanne Haviland

Benjamin R Hawkins

Maria Hawkins

Paulina Hawkins

Kentaro Hayashida

Lawrence Hayes

Yulei He

Xavier A Hébuterne

Tobias Hecker

Manfred Hecking

Nina Heinrichs

Hans Heinzer

Lucie Heinzerling

Jonas Helgertz

Karin Hellner

Christian Hellum

Olli Helminen

Joris Hemelaar

Vincent Hendriks

Niel Hens

Maria Trinidad Herrero

Elke Hertig

Katharina Hess

Marjolein Heuvelmans

Mark Hew

Andrew Hill

Kate Himmelmann

Elif Hindié

Rosemary Hiscock

Lars Hjorth

Valentina Hlebec

Zoë S J Hoare

Barbara Hoffmann

Charles W Hoge

Chris Holden

Loes Hollestein

Mette Holst

Rosemary Horne

Seyed M Hosseini-Moghaddam

Louise Howard

Jessica Howell

Daniel J Hruschka

Htet Lin Htun

Veronika Huber

Jennie Hudson

Michael Hultström

Christian Victor Hulzebos

Nicole Hunfeld

Nusrat Husain

Sharon J Hutchinson

Jane Hutton

Lars Christer Hydén

Mari Hysing

Licia Iacoviello

Antonio Iannelli

Pedro Iglesias

Sindana Ilango

Irena M Ilic

Jorma Ilonen

Suzanne Ingle

Bruce Innis

Ana-Maria Iosif

Stephanie Irving

Nigel Irwin

Takeaki Ishizawa

Andrea Isidori

Zahra Jafari

John Michael Jakicic

Dejan Jakimovski

Janet Janbek

Joseph A M J L Janssen

Geert O Janssens

Luis Jara-Palomares

Channa Jayasena

Helena C B Jernström

Jianping Jia

David Jiménez-Pavón

Michael Joannidis

Ane Johannessen

Gudmundur Johannsson

Anna Johansson

Catherine Johnson

Vilija Jokubaitis

Stefán Jónsson

Deepak Joshi

Bastien Joubert

Anna Jöud

Mariona Jové

Steven Julious

Markus Juonala

Sandra Juul

Andre Kalil

Tomas Kalincik

Zoltan Kalo

Mona Kanaan

Ad Kaptein

Christian Karagiannidis

Mohammad Karamouzian

Thomas Karlas

Özge Erişöz Kasap

Stefan Kasper

Karin Kasza

Jakob Kather

Srinivasa Katikireddi

Taigo Kato

Hugo A Katus

Verena Andrea Katzke

Howard Kaufman

Pamela Kearns

Mark Kelson

Zoe Kelson

Line Kenborg

Eilis Kennedy

Nicholas Kennedy

Kelly M Kenzik

Jim S Khan

Ali Khashan

Kamlesh Khunti

Arif Khwaja

Eliud Kibuchi

Berit Kieselbach

Carolin Kilian

Alfred Kim

Yong-Bae Kim

Vasilios Κ Kimiskidis

Kaan Kirali

Julien Kirchgesner

Heinz-Josef Klümpen

Michael Knipper

Johannes Knitza

Ann Kristin Knudsen

Robin Köck

Klaus Kofoed

Jussi Koivunen

Niels Kok

Kaija-Leena Kolho

Loreta A Kondili

Evangelos Kontopantelis

Dirk Korf

Janne Koskimäki

Karel Kostev

Konstantinos Kostikas

Nikolaos Koutsouleris

Justyna Kowalska

Liubov Kozlovskaya

Shane W Kraus

Felix Krenzien

Peter Lommer Kristensen

Jesper Krogh

Theocharis Kromydas

Andi Krumbholz

Eva Kubala Havrdová

Ralf Kuhlen

Hannah Kuper

Margaret K Kyle

Taina Laajasalo

Tiina K M Laatikainen

Anna Lam

Stephen Lam

Vincent Wai To Lam

Marcello Lanari

Rikard Landberg

Giovanni Landoni

Douglas John Lanska

Sophie M Lanzkron

Therese Sophie Lapperre

Thomas Larsen

Elin Larsson

Annmarie Touborg Lassen

Aurélie Lasserre

Kathrin Lauber

Jeffrey Lazarus

Romain Lazor

Carel le Roux

Thierry Le Tourneau

Marto Leal

David Lee

Eudocia Lee

Ji-won Lee

Shaun Lee

Wade Lee-Smith

Jiayao Lei

Daniel Leightley

Cédric Lemogne

Marco Lenti

Iracema Leroi

Philippe Lesprit

Janni Leung

Yvonne Leung

Emily B Levitan

Sonia Lewycka

Bénédicte Leynaert

Huiqi Li

David Liebeskind

Cherry Lim

Chloe Lim

Youn-Hee Lim

Mario Lima

Ashley Linden-Carmichael

Suping Ling

Hartmut Link

Lynda Lisabeth

Li Liu

Yuewei Liu

Michael Livingston

Plácido Llaneza

Elba Llop

Carl Llor

Judith Löffler-Ragg

Jose Lopez-Miranda

Catherine Lord

Cesaltina Ferreira Lorenzoni

Johannes Lorscheider

Valérie Louis

Hugo Lövheim

Marianne Lovstad

Mario Luciano

Ramon Luengo-Fernandez

Åke Lundkvist

Georg Lurje

Andrés Chamarro Lusar

Amanda Ly

Frances Lynch

Elsebeth Lynge

Ya'nan Ma

Graeme MacLennan

Janet MacNeil Vroomen

Sten Madsbad

Michael Maeng

Laura Magee

Nicos K Maglaveras

Dianna Magliano

Manuel Maglione

Viyaasan Mahalingasivam

Cristiane Maia

Marsha R Mailick

Mario M Maj

Govind Makharia

Konstantinos Makris

Umberto Malapelle

Rainer Malik

Kenneth Man

Ranjit Manchanda

Emmanuel Mandonnet

Olivia Manfrini

Gregory Mann

Mohammad Ali Mansournia

Jakob Manthey

Stéphane Manzo-Silberman

Stefan J Marciniak

Hani Joseph Marcus

Sylvestre Maréchaux

Braulio Marfil-Garza

Giovanni Marini

Daniela Mariosa

Lauri Markowitz

Michael Marks

Suzanne Marks

Robby Markwart

Mariano Marrodán

Maria Marti-Castaner

Cara Martin

Esteban Martinez

Mario Martinez Jimenez

Eugenia Martinez-Hernandez

Manuel Martinez-Selles

Pierre L Martin-Hirsch

Neil Martinson

Grace Marx

Deborah Mascalzoni

Pierre Masselot

Lourdes Mateu

Koji Matsuo

Anna Vittoria Mattioli

Soeren Mattke

Konstantinos Mavridis

Julien Mazières

Marianna Mazza

Andrew McAuley

Naomi McCallion

Colm McDonald

Emily McDonald

Linda K McEvoy

Bradley McGregor

Stuart McIntosh

Meredith McMorrow

Gillian Mead

Benjamin Meder

Anurag Mehta

Ella Mendelson

Shanthi Mendis

Fiona Mensah

Sebastiano Mercadante

Guillaume Meric

Tracy L Merlin

Karen Messing

Niina Metsä-Simola

Patrick Meybohm

Gerd Meyer zu Hörste

Daniel Michaeli

Laure Michel

Sarah Michiels

Rafael Mikolajczyk

Kenneth Miller

Mark Miller

Tatsuya Mima

Sanjana Mitra

Babak Moazen

Joerg Moehrle

Markus Moessner

Pasquale Mone

Claudio Montalto

Fabrizio Montarsi

Emmanuel Montassier

Ali Montazeri

Catrin Moore

Enrique Morales

Lina Morch

Maria Cristina Morelli

Lucas Moreno

Meg Morris

Orla Morrissey

Bente Morseth

Laust Hvas Mortensen

Giovanni Mosiello

Enrico Mossello

Elias Mossialos

Sergey Motov

Ruben Dario Motrich

Sheena Mukkada

Judy R Mullan

Thomas Jörg Müller

Monica Mullin

Praveen V Mummaneni

Darren M Mylotte

Ramesh Nadarajah

Mitsutoshi Nakada

Atsushi Nakajima

Indra Narang

Jean–Charles Nault

Edita Navrátilová

Valentina Nekljudova

Randy J Nelson

Ursula Nestle

Edmond Ng

Nhung Nguyen

Tuan Anh Nguyen

Federico Nichetti

Claire Niedzwiedz

Peter Nielsen

Teemu Niiranen

Vladyslav Nikolayevskyy

Dorothea Nitsch

Riccardo Nocini

Axel Nordenskjöld

Bo Nordenskjöld

Marina Noris

John Norrie

Kate Northstone

Therese Haugdahl Nøst

Saad Nseir

Marcio Nucci

Alistair Victor William Nunn

Erfan Nur

Tommy Nyberg

Malin Nygren-Bonnier

Jaana Oikkonen

Shalini Ojha

Melissa Oldham

Kevin Kris Warnakula Olesen

Morten Salling Olesen

Timothée Olivier

Raffaele Ornello

Corentin Orvain

Megan Othus

Naïm Ouldali

Alice Owen

Julianna Pacheco

Andrzej Pająk

Lasse Pakanen

Amir Haji Agha Pakpour

Ståle Pallesen

Diego Palumbo

Daniel Pan

Pasquale Paolisso

Sergi Papiol

Elizabeth Davida Paratz

Milosz Parczewski

Manish Pareek

Jean-Jacques Parienti

Alexander A Parikh

Elyse Richelle Park

Jakob Passweg

Susanne Paukner

Friedemann Paul

Maria Pavia

Piero Pavone

Marc G Pawlitzki

Shlomit Paz

Lillian Pazvakawambwa

Jonathan Pearson-stuttard

Markus Peck-Radosavljevic

Carsten Bøcker Pedersen

Ulrik Pedersen-Bjergaard

Joel R Pekow

Einat Peles

José L Peñalvo

Nicolas Penel

Thomas Penzel

Francesco Pepe

Jose Perea

Subha Perni

Ferenc Peták

Gunnel Peterson

Donatella Petretto

Nicola Petrosillo

Sean Phelan

Pedro Piedra

Daniela Pierannunzio

Maria Catherine Pietanza

Olivier Piguet

Matthias Pinter

Snehal Pinto Pereira

Francesco Pistelli

Constantinos Pitsios

Davor Plavec

Mathias Pletz

Dimitri Poddighe

Paul Poirier

Piero Poletti

Andrea Poli

Olli Polo

Caridad Pontes

Carolina Porras Gutiérrez

Camillo Porta

Anja Poulsen

Ferran Prados

Robin Prescott

Mariano Provencio

Rui Providência

John Puntis

Katri Pylkäs

Manuela Rabaglio

Nicole Racine

Francesco Raimondi

Giovanni Raimondo

Gita Rajan

Emanuel Raschi

Aidin Rawshani

Jürgen Rehm

Ihtesham Rehman

Željko Reiner

Ana Requena-Mendez

Vincenzo Restivo

Uwe Reuter

Isabela Ribeiro

Walter Ricciardi

Michael A Richards

Debra Richardson

Matteo Richiardi

David Rimmele

Annapaola Rizzoli

John D Roache

Graham Roberts

Jo Robinson

Joacim Rocklöv

Carlos Rodrigo

Fernando Rodriguez-Artalejo

Bart O Roep

Martin Roland

Christian Rolfo

Andres Roman-Urrestarazu

Manuel Romero-Gomez

Cecile Ronckers

Robin Room

Jutta Roosen

Martin Röösli

Maria Rosa

Rafael Rosell

Peter Rowe

Dana Rubenstein

Anthony Rudd

Shivaprakash Rudramurthy

Hans Jürgen Rumpf

Martin Rutegård

Mark Rutherford

Erich Rutz

Dermot Ryan

Patrick Ryan

Anum Saeed

Hajirah Saeed

Umar Saeed

Marc Saez

Moahmmad Ali Sahraian

Yoshihiro Sakamoto

Faouzi Saliba

Raimo Salokangas

Cristina Sampaio

Olivier Sanchez

Pablo José Sánchez

Gabriele Sani

Eugenio Santoro

Gaetano Santulli

Rosario Sarabia

Shiv Sarin

Naveed Sattar

Alessia Savoldi

Andrea Schaffer

Francis Schaffner

Alejandro Schijman

Maarten Schim van der Loeff

Moritz Schmelzle

Rebecca Schmidt

Matthias Schulze

Walter Joachim Schulz-Schaeffer

Robert Schwartz

Roberto Scicali

Katrina Scior

Jonathan Scourfield

Nick Sculthorpe

Sarah Seaton

David Sebag-Montefiore

Anna Lene Seidler

David Seiffge

Kevin J Selby

Johann Sellner

Thiviya Selvanathan

Erin O'Brien Semmens

Victor L Serebruany

Raphaele Seror

Ary Serpa Neto

Laurent Servais

Belgin Sever

Andrea Severino

Aref Shariati

Michelle W Sharp

Daisy Shepherd

Lianne Sheppard

Satoshi Shiono

Aron R Shlonsky

Lester M Shulman

Spyridon Siafis

Volkert Dirk Siersma

Peter Siesjö

Catarina Simões Padilla

Jacqueline Sin

Julia Sinclair

Anika Singanayagam

Daisy Singla

Zbigniew Siudak

Ieva Skarda

Nicole Skoetz

Barbro Skogman

Audrey Smargiassi

Nynke Smidt

Henry Smith

Letícia Soares

Ole Søgaard

Tore Solberg

Johan Nilsson Sommar

Iris Sommer

Milan Sonneveld

Joan Soriano

Vicente Soriano

Savino Spadaro

Ernesto Sparrelid

Katie Spencer

Ute Spiekerkoetter

Vatsala Rangachar Srinivasa

Alexander Stahmann

Margaret Anne Stanley

Norbert Stefan

Tilman Steinert

Ruth Stephen

Andrew Steptoe

Evie Stergiakouli

Robert Stewart

Katharina Stibrant Sunnerhagen

Eric Stice

Heidi Stöckl

Neil Stone

Ketil Stordal

Henrik Støvring

Luc Strobbe

Dominik Sturm

Marc Suárez-Calvet

Maria Sundaram

Robert Sutcliffe

Matt Sutton

Steven Sutton

Teodor Mikael Svedung Wettervik

Jannet Svensson

Tamas Szakmany

Silvio Tafuri

Julien Taieb

Emanuela Taioli

Iztok Takač

Keisuke Takano

Kinaciyan Tamar

Xiao Tan

Julian Tang

Shao-tao Tang

Pieter Tanis

Ignacio Tapia

Florin-Andrei Taran

Jose V Tarazona

Davide Tassinari

Maria-Luisa Tataranno

Magdalena Taube

Vicky Taxiarchi

Awoke Temesgen

James Teo

Takeshi Terao

Masanori Terashima

Katja Tervahartiala

Bianca Tesi

Inge Tetens

Stephanie Thomas

Christoph Thomssen

Pernille Thrane

Petra Thürmann

Anders Tingberg

David C Tipene-Leach

Claudio Tiribelli

Pierre Tissières

Vivianne Tjan-Heijnen

Andrew Tonkin

Aleksandra Torbica

Antoni Torres

Claudia Torres-Farfán

Patrick Tounian

Seray Töz

Hadrien Tranchart

Dario Trapani

Jonel Trebicka

Giusto Trevisan

Adam Trickey

Anjan Trikha

Pelagia Tsoutsou

Patricia Tucker

Jaakko Tuomilehto

Anthony Turpin

Peter Tyrer

Jacob Udell

Obioha Ukoumunne

Anand Vaidya

Marleen Van Baak

Bert Jan H van den Born

Ann Van den Bruel

Lambert van den Heuvel

Henriette E van der Horst

Jan van der Meulen

Marc van der valk

Hans van der Wouden

H Rogier van Doorn

Nick van Es

Willem A Van Gool

Jony van Hilst

Folkert van Kemenade

Jan Van Meerbeeck

Rick van Rijn

Ard van Sighem

Rama Vancheeswaran

Eugeen Vanmechelen

Sven Vanneste

Gianluca Vanni

Tommi Juhani Vasankari

Marc Vasse

G D Marijn Veerman

Galina Velikova

Aurélien Venara

Amy C Verdun

Lasse Vestergaard

Peter Vickerman

Ramon Vilallonga

Carmen Vinaixa

Jean-Louis Vincent

Marco Vinceti

Michelle M Vine

Vasiliy Vlassov

Melandri Vlok

Judith M Vonk

Jon Wadsley

Kayo Waki

Alexander Walley

David Walsh

Martin Walter

Emily S Wan

Per Wandell

Duolao Wang

Han-I Wang

Yanzhong Wang

Ahmed Waqas

Allison Waters

Helen Weiss

Stephanie Weiss

Stefan Welter

Jinglin Wen

Sara Wen

Martin Wermke

Marten Werner

Virginie Westeel

Peter Westerweel

Rene Westhovens

Björn Wettermark

Jack Wetzels

Steven Wexner

James White

Richard Whittington

Heinz S Wiendl

Maarten Wijnenga

Parke Wilde

Peter Willatts

Monnica Williams

Thomas Ashby Wills

Katharina Wimmer

Paul Y Windisch

Martha Wingate

Bo Gregers Winkel

Anna Winkvist

Petra Woestenberg

Frank Wolters

Rebecca Wong

Robert Wong

Vincent Wong

Stephen L Wood

Kate A Woodcock

Angela Woods

Jordan Wright

Qi-Jun Wu

Vin-Cent Wu

Ningshao Xia

Tong Xia

Ying Xiao

Yan Xie

Zhiwei Xu

Sıddika Songül Yalçın

Huanming Yang

Ting Yang

Keith Yeates

Juan José Yepes-Nuñez

Changhoon Yoo

Gilbert Youssef

Ke-da Yu

Oriana Hoi Yun Yu

Rongqin Yu

Erlangga Yusuf

Alexander Zarbock

Hajo Zeeb

Alexei Zelenev

Jean Pierre Zellweger

Henrik Zetterberg

Líbia Zé-Zé

Fengqing Zhang

Hailiang Zhang

Lei Zhang

Yuqing Zhang

Fengmin Zhao

Peng Zheng

Youbin Zheng

Feng-Cai Zhu

Wendy Ziai

Martha Zimmermann

Scott Zuckerman

Daniele Zullino

Daniel Zwahlen

